# Supplementary material for: Characterizing Circulating Tumor Cells and Tumor-Derived Extracellular Vesicles in Metastatic Castration-Naive and Castration-Resistant Prostate Cancer Patients
Source: Cancers (Basel). 2022 Sep 10;14(18):4404. doi: 10.3390/cancers14184404 (PMC9497200; doi:10.3390/cancers14184404)
Supplement: Supplementary file 1 [file cancers-14-04404-s001.zip › cancers-1867067-supplementary.pdf]

Table S1. Spearman Rho test - correlation of CTCs and CTC sunglasses to tdEVs in CNPC and CRPC.

CNPC patients			CRPC patients		
Correlations with tdEVs_ACCEPT	Spearman Rho coefficients	*p* - value	Correlations with tdEVs_ACCEPT	Spearman Rho coefficients	*p* - value
CTC_Cluster	0.56	< 0.001	CTC_Cluster	0.78	< 0.001
CTC_Pretty	0.68	< 0.001	CTC_Pretty	0.69	< 0.001
CTC_Heterogenous_CK	0.81	< 0.001	CTC_Heterogenous_CK	0.86	< 0.001
CTC_Cleaved_CK	0.67	< 0.001	CTC_Cleaved_CK	0.82	< 0.001
CTC_Fragmented_DNA	0.57	< 0.001	CTC_Fragmented_DNA	0.55	< 0.001
CTC_Cleaved_Fragmented	0.66	< 0.001	CTC_Cleaved_Fragmented	0.66	< 0.001
CTC_ACCEPT_Corrected	0.84	< 0.001	CTC_ACCEPT_Corrected	0.92	< 0.001

Table S2. CTC and tdEV counts from manual, ACCEPT and ACCEPT corrected analysis.

		**CNPC (n = 104)**			**CRPC (n = 66)**			
		**0 CTC**	**1 ≤ CTC < 5**	**CTC ≥ 5**	**0 CTC**	**1 ≤ CTC < 5**	**CTC ≥ 5**	
		Range (Mean, SD)	Range (Mean, SD)	Range (Mean, SD)	Range (Mean, SD)	Range (Mean, SD)	Range (Mean, SD)	
CTC	manual	-	1–4 (1.8, 0.9)	5–1903 (163, 333)	-	1–4 (2, 1.14)	5–378 (62.6, 83.4)	
tdEV	ACCEPT	0–49 (5.79, 7.40)	1–252 (30.33, 47.59)	8–3373 (657, 915)	0–12 (5, 4)	2–138 (34.31, 33.86)	28–2196 (409, 547)	p = 0.005 p = 0.937 (CTC = 0) p = 0.3) (1 ≤ CTC < 5) p = 0.806 (> 5 CTC)
CTC	ACCEPT	-	1–4 (1.92, 0.96)	5–1046 (77, 150)	-	1–4 (2.37, 1.02)	2–252 (45.82, 56.03)	p = 0.011
CTC	ACCEPT corrected	-	1–4 (1.92, 0.95)	5–851 (107, 178)	-	1–4 (1.93, 0.92)	5–229(37.93, 48.93)	p = 0.013 p = 0.888 1 ≤ CTC < 5 p = 0.146 (> 5 CTC)

Table S3. CTC subclasses (%) in CNPC and CRPC patients. .

	**CNPC (n = 104)**		**CRPC (n = 66)**		
	**1 ≤ CTC < 5**	**CTC ≥ 5**	**1 ≤ CTC < 5**	**CTC ≥ 5**	
	% of CTC Range, mean, SD	% of CTC Range, mean, SD	% of CTC Range, mean, SD	% of CTC Range, mean, SD	
CTC clusters	3.84% 0–1 (0.07,0.26)	3.97% 0–49 (4.27, 10.09)	0	6.01% 0–8 (2.28, 2.34)	p = 0.006
Pretty CTCs	9.61%	24.95%	9.67%	27.34%	p = 0.069

	0–1 (0.18, 0.39)	0–201 (26.82, 48.89)	0–1 (0.18, 0.40)	0–89 (10.37, 21.84)	
Heterogenous CTC	61.53%<br>0–3 (1.18, 0.96)	47.22%<br>3–598 (50.75, 111)	67.74%<br>0–4 (1.31, 1.3)	40.60%<br>3–45 (15.40, 11.89)	p = 0.041
Cleaved CK	15.38%<br>0–2 (0.29, 0.54)	14.95%<br>0–160 (16.06, 34.56)	6.45%<br>0–1 (0.12, 0.34)	15.81%<br>0–60 (6, 12.81)	p = 0.052
Fragmented DNA/CK	1.92%<br>0–1 (0.03, 0.19)	1.99%<br>0–15 (2.14, 3.8.4)	3.22%<br>0–1 (0.06, 0.25)	3.29%<br>0–13 (1.25, 2.98)	p = 0.349
Cleaved CK + Fragmented DNA	7.69%<br>0–1 (0.14, 0.36)	6.89%<br>0–71 (7.41, 14.48)	12.90%<br>0–1 (0.25, 0.44)	6.91%<br>0–20 (2.62, 4.72)	p = 0.357
